# Heat Shock Protein 70 and 90 Family in Prostate Cancer

**DOI:** 10.3390/life12101489

**Published:** 2022-09-26

**Authors:** Xun Fu, Jiang Liu, Xin Yan, Michael E. DiSanto, Xinhua Zhang

**Affiliations:** 1 Department of Urology, Zhongnan Hospital of Wuhan University, Wuhan 430000, China; 2 Department of Surgery and Biomedical Sciences, Cooper Medical School of Rowan University, Camden, NJ 08028, USA

**Keywords:** heat shock protein, HSP70s, HSP90s, prostate cancer

## Abstract

Prostate cancer (PCa) is the second most frequent cancer that affects aging men worldwide. However, its exact pathogenesis has not been fully elucidated. The heat shock protein (HSP) family has cell-protective properties that may promote tumor growth and protect cancer cells from death. On a cellular level, HSP molecules have a strong relationship with multiple important biological processes, such as cell differentiation, epithelial–mesenchymal transition (EMT), and fibrosis. Because of the facilitation of HSP family molecules on tumorigenesis, a number of agents and inhibitors are being developed with potent antitumor effects whose target site is the critical structure of HSP molecules. Among all target molecules, HSP70 family and HSP90 are two groups that have been well studied, and therefore, the development of their inhibitors makes great progress. Only a small number of agents, however, have been clinically tested in recruited patients. As a result, more clinical studies are warranted for the establishment of the relationship between the HSP70 family, alongside the HSP90 molecule, and prostate cancer treatment.

## 1. Introduction

Prostate cancer (PCa) is the second most frequently diagnosed cancer in men worldwide, with an incidence of 14.1% and a mortality of 6.8% in 2020 [1]. The incidence rate of PCa has been increasing in the vast majority of countries over recent decades, while death rates have varied greatly worldwide (up to 10-fold) [2]. Despite its high incidence and mortality, the complexity of PCa pathogenesis makes it difficult to offer a radical treatment for PCa patients. Growing evidence has been published to elucidate its pathogenesis associating with androgen/androgen receptor (AR), estrogen, genetic changes, alterations of signal pathways, inflammation and infections, leptin and so on [2]. More mechanistic studies are still necessary.

**Figure 1:**
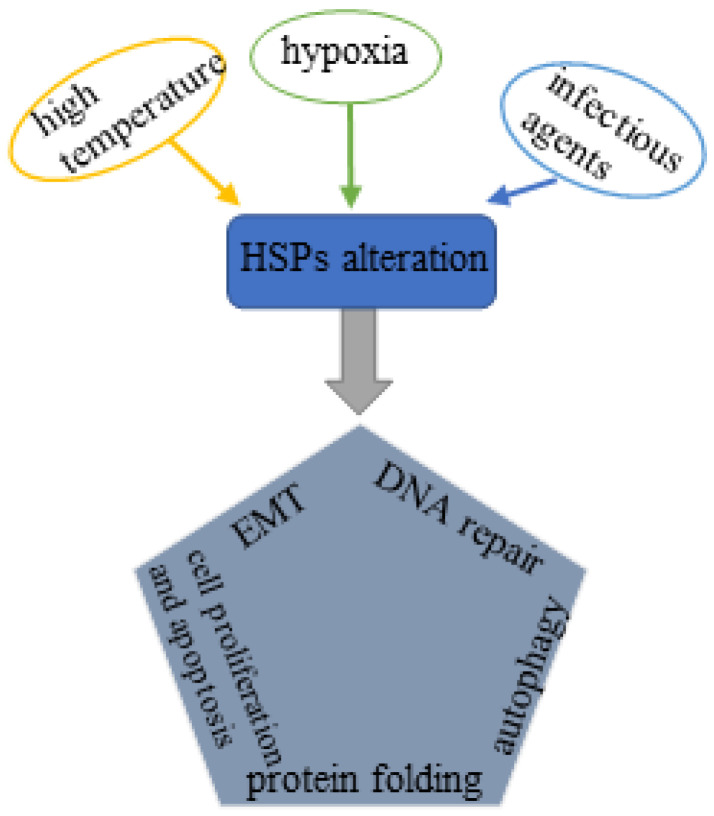
Schematic illustration of the influencing factors of HSPs alteration and its relationship with PCa pathogenesis: External stresses such as hyperthermia, hypoxia and infectious agents stimulate the expression of HSPs, which further affects protein folding, pyroptosis and apoptosis, autophagy, EMT, and DNA repair and ultimately triggers the pathophysiological process of prostate cancer.

Heat shock proteins (HSPs) are conserved molecular chaperones that have cytoprotective properties and are upregulated in response to multiple pathophysiological stresses induced by extensive stimulations, such as high temperature, hypoxia and infectious agents (bacterial and viral) [3]. HSPs are categorized into six subfamilies based on the molecular weight: HSP110, HSP90, HSP70, HSP40, small HSPs, as well as chaperonin families [4]. Among all these HSPs, HSP70s (the HSP70 family) and HSP90s (the HSP90 family) are the most two well-studied molecular families with multiple functions. HSP70s are composed of 13 members principally including inducible HSP70 (HSP72 or HSPA1), constitutive heat shock homologous protein 70 (HSC70), glucose-regulated protein 78 (GRP78) and mortalin (GRP75) [5]. HSP90s consist of four members: two in cytosol (HSP90AA1 and HSP90AB1), one in endoplasmic reticulum (ER) (GRP94, also called HSP90B1) and one in mitochondria (TRAP1) [5,6]. The HSP70 and HSP90 family members share the similar regulatory pattern that both require co-chaperones to regulate their functional cycles [7]. These two molecular families are thought to serve as housekeeping genes with basic expression levels under normal conditions; upon exposure to the external stress factors, they are overexpressed to fight against the stressed response. Accumulating evidence has paid attention to the involvement of HSP70 and HSP90 molecular family in multiple biological processes related to tumorigenesis, such as tumor growth, invasion, metastasis. On a cellular level, HSP70s and HSP90s are involved in chaperone activity, protein folding, cell survival and proliferation, cell apoptosis, autophagy, cell differentiation, epithelial–mesenchymal transition (EMT), fibrosis, DNA repair, etc. [8,9,10,11,12] (Figure 1), contributing to the initiation, development and progression of prostate cancer. Based on the functions of HSP70s and HSP90s in tumorigenesis, a number of agents targeting the critical structure of two molecular families have been successfully developed. These developed agents are divided into three categories: molecular inhibitors, aptamers and immune biologics: antibodies and vaccines. However, only a small number of agents have been subjected to the clinical trial with only three agents tested in recruited PCa patients. In this review, we focus on the important role of HSP70s and HSP90s in PCa pathogenesis. We also highlight the potential value of two molecular families as therapeutic targets in PCa treatment.

## 2. Prostate Cancer

The malignant transformation of the prostate follows a multi-step process, first prostatic intraepithelial neoplasia (PIN), then localized prostate cancer, followed by locally infiltrated advanced prostate adenocarcinoma, and finally leading to metastatic prostate cancer [13]. The etiology of prostate cancer is complicated and has not been fully elucidated. Known etiologies of prostate cancer include but are not limited to androgen/androgen receptor (AR), EMT, genetics, and pathway alterations.

Androgens regulate the development, maturation, and maintenance of the prostate and affect the proliferation and differentiation of the luminal epithelium. Androgen exposure plays an important role in the occurrence and maintenance of prostate cancer. AR is expressed in almost all primary and metastatic PCa, most of which initially respond to androgen deprivation. Androgen deprivation can improve the prognosis of prostate cancer patients, enabling reduced metastasis and prolonged survival [2]. Thompson and his colleagues treated subjects with finasteride (one of the 5α-reductase inhibitors (5ARI)) and placebo, observing that the incidence rate of prostate cancer was significantly lower in the experimental group than in the control group. 5ARI can reduce the incidence rate of prostate cancer by 24.8% by inhibiting the conversion of testosterone (T) into more effective dihydrotestosterone (DHT) [14].

Normally, the epithelium is a monolayer tightly held by adhesive proteins, and this immobilization prevents movement of cells from the monolayer [15]. In the course of PCa development, epithelial cells are thought to undergo EMT process. It is shown that epithelial cell markers (such as E-cadherin and ocludins) are downregulated, resulting in loss of cell adhesion, while mesenchymal markers (such as vimentin and N-cadherin) are upregulated, thereby allowing tumor cells to migrate or transfer to different organs [16]. Furthermore, growth factors and cytokines, such as transforming growth factors β (TGF- β) [17], epidermal growth factor (EGF) [18], and insulin-like growth factor (IGF) [19], as well as signaling pathways, such as mitogen activated protein kinase (MAPK) and phosphatidylinositol 3-kinase (PI3K) [20], are able to regulate the expression of E-cadherin, N-cadherin and ZEB1 and therefore influence EMT levels, which ultimately affects the progression of prostate cancer. This indicates that EMT is essential in the occurrence and metastasis of prostate cancer.

A large number of studies have shown that genetics plays a critical role in the initiation and development of prostate cancer. In the mid-20th century, Woolf et al. [21] published a report on family aggregation for the first time. This observation was supported by subsequent case–control and cohort studies [22]. Zeegers et al. [23] conducted a meta-analysis on 33 epidemiological studies of prostate cancer and found that relative risk was 2.53 (95% confidence interval, 2.24–2.85) for first-degree family members. The relative risk increases with the number of affected family members, degree of relationship, and age. In addition, the alterations of gene expression are also considered to be related to the development of prostate cancer, such as *HOXB13, BRCA1, BRCA2,* as well as our protagonists *HSP90* and *HSP70* [24,25,26,27]. In an in vitro experiment, *HOXB13* and *MEIS1* interact to regulate the proliferation and apoptosis of multiple prostate cancer cell lines (DU145, E006AAT, PC3, CWR22RV1, LNCaP, LNCaP C4-2, and VCap) [28], which is believed to regulate the development of prostate cancer. Early studies showed that *BRCA1* and *BRCA2* mutations have an increased risk of prostate cancer. It is estimated that *BRCA1* mutations increase prostate cancer risk by 1.8 to 3.5 times and *BRCA2* by 4.6 to 8.6 times. *BRCA*-related prostate cancer is more likely to metastasize and deteriorate in the late stage [29,30]. As for HSP70 and HSP90 molecules, we will focus on them in the following section.

## 3. HSP Family

### 3.1. HSP70 Family

As the most important member in the whole HSP family, HSP70s are upregulated in response to various cellular stresses and able to protect cells from fatal damage [31,32]. HSP70 family molecules have been shown to be involved in cell apoptosis, proliferation, and differentiation. Human HSP70s are widely expressed in a wide range of cell components (such as ER, proteasome, ribosome, mitochondria, and lysosomal membrane). It is believed that the expression of HSP70s is increased in many cancers, such as PCa, breast cancer, which may be associated with the tumorigenesis mechanism [33]. In patients with PCa, serum levels of HSP70s were significantly higher than in patients without PCa [34]. In addition, castration resistant prostate cancer (CRPC) is associated with increased reliance on HSP70s [35], and GRP78 expression is significantly elevated in metastatic CRPC compared with localized prostate cancer [36]. Moreover, GRP75 expression correlates with increased risk of high-grade prostate adenocarcinoma [37]. However, a report published in 2000 indicated that the expression of HSP70 molecules was unaltered in early prostate cancers but was reduced in morphologically advanced cancers compared with non-neoplastic prostate epithelium [38]. These findings suggest that HSP70s may serve as a diagnostic indicator and prognostic indicator for PCa.

#### 3.1.1. Structure of HSP70

**Figure 2:**
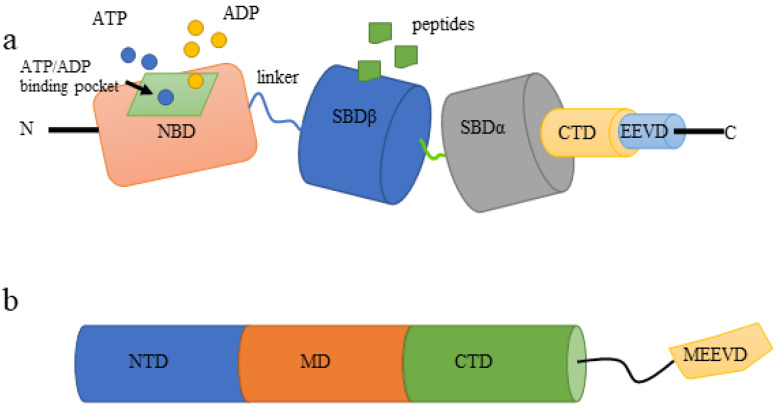
Structure of HSP70s and HSP90s (**a**) HSP70s consist of two main domains, namely, the N-terminal nucleotide-binding domain (NBD) responsible for ATPase activity and the C-terminal substrate-binding domain (SBD) required for peptide binding. The linker connects two domains. The N-terminal NBD provides ATP/ADP pockets for ATP binding. SBD is further divided into two subdomains (SBDα and SBDβ) and followed by a C-terminal ending with an EEVD motif. The main function of SBDβ is to bind substrate peptides, which is the critical step of substrate folding. (**b**) HSP90s monomers are composed of an amino terminal domain (NTD), an intermediate domain (MD), and a C-terminal domain (CTD). CTD contains a C-terminal met-Glu-Glu-Val-Asp (MEEVD) motif. The three domains function differently: the NTD mediates binding to ATP, the MD is involved in ATP hydrolysis and HSP90 binding to client proteins and the CTD is responsible for the formation of dimers.

The HSP70 family is highly conserved in evolution and consists of two major domains. One is the N-terminal nucleotide-binding domain (NBD) responsible for ATPase activity, and the other is the C-terminal substrate-binding domain (SBD) required for peptide binding. The two domains are connected by a linker (Figure 2a). The N-terminal NBD provides ATP/ADP pockets for ATP binding, which is critical for the ATPase reaction that is required for protein folding and release [8]. HSP70 binding protein 1(HSPBP1) and Bcl2-associated athanogene 1 (BAG-1), the functional orthologous nucleotide exchange factors of HSP70s, catalyze the release of ADP from HSP70s while inducing different conformational changes in the ATPase domain of HSP70s. An appropriate exchange rate of ADP/ATP is crucial for the chaperone dependent protein folding process [39]. SBD is further divided into two subdomains, namely SBDα and SBDβ, and followed by a C-terminal domain (CTD) that ends with an EEVD motif. The subdomain SBD β is a peptide binding capsule with which peptides can be bound as substrates. The binding and dissociation of substrates are necessary for HSP70 family members to fold substrates. Allosteric effects between functional domains modulate the ATPase cycle, in turn, polypeptide uptake and release by HSP70s [8,40].

#### 3.1.2. HSP70s and AR

HSP70s can maintain the androgen receptor (AR) inactive phase [41]. These chaperones are released in the presence of androgen, prompting transactivation and receptor clustering. Previous studies showed that HSP70s, assisted by its co-chaperone HSP40, are able to recognize a region of the AR named N-terminal domain (NTD) including the FQNLF motif. This motif activates AR by interacting with the AR ligand binding domain (LBD). This suggests that HSP70s and the LBD compete for the FQNLF motif and in turn regulate AR activation [41]. In addition, HSP70s are molecular chaperones for AR variants lacking the LBD domain, ensuring their stability and function in cooperation with HSP40 molecule, which is disadvantageous in the treatment of CRPC patients [42]. These findings suggest that inhibition of HSP70s may suppress the promotive effects of androgens on PCa. Dong et al. demonstrated that the HSP70 molecule is a cofactor of the N-terminal domain (NTD) of AR in prostate cancer cells. HSP70 inhibition by siRNA significantly downregulated the expression of endogenous AR and concomitantly suppressed the transcriptional activity of AR. This suggests that HSP70s play an important role in AR activation [24]. Proteostasis is a potential mechanism that contributes to cancer cell survival and drug resistance. The proteostasis of AR/AR-V7 can be maintained by the interaction of STUB1, a ubiquitin ligase, with HSP70. STUB1 dissociates AR/AR-V7 from HSP70, leading to AR/AR-v7 ubiquitination and degradation. Inhibition of HSP70 significantly inhibits prostate tumor growth and improves anti androgen therapy in CRPC via AR/AR-V7 inhibition. Clinically, HSP70 expression is upregulated and correlates with AR/AR-V7 levels in high Gleason score PCa, suggesting that HSP70 may be a predictor of androgen dependent prostate cancer [43]. Moreover, the dynamic composition of the AR folding complex and AR function are influenced by BAG-1M and HSPBP1. As HSPBP1 and BAG-1 protein levels increase, AR function is inhibited [39].

#### 3.1.3. HSP70s and Cell Apoptosis and Proliferation

In mammals, alterations in HSP70s levels significantly affect cell apoptosis and proliferation. Mammalian sterile 20-like kinase 1 (Mst1) is a ubiquitously expressed serine/threonine kinase whose activation leads to apoptosis. HSP70 overexpression mediated the degradation of Mst1in the proteasome-dependent pathway, inhibiting the proapoptotic effect of Mst1, which in turn suppresses the apoptosis in two prostate cancer cell lines: LNCaP and DU145 [44]. A study by Gibbons et al. [45] also showed that HSP72, a member of the HSP70 family, could inhibit apoptosis of prostate tumor cells through the Mcl-1, Bcl-2, Bcl-X (L), and glutathione-S-transferase (GST) pathways. Jones et al. found that HSP72 directly inhibited apoptosis in a dose-dependent manner. Depletion of HSP70 using antisense oligonucleotides against HSP70 mRNA or the bioflavonoid drug quercetin can lead to tumor cell PC-3 apoptosis in the absence of stress [46]. HSP70s bind and fold newly synthesized cyclinD1 and are involved in the assembly of cyclinD1/cyclin-dependent kinase 4 (CDK4). CyclinD1 is amplified and overexpressed in many cancers. Cyclin D1/CDK4/6 holoenzyme complex is stimulated by the mitotic signal cascade to accelerate the proliferation of cancer cells [47].

#### 3.1.4. HSP70s and EMT, Migration, Invasion, and Metastasis

Previous studies have confirmed that HSP70s play a key role in tumor EMT, invasion, migration, and metastasis in vitro, including but not limited to lung cancer [48], colorectal cancer [49], glioblastoma [50], breast cancer [51], and Pca [52,53]. Cultrara et al. knocked down GRP78 in PC3 cells, which in turn reduced the expression of E-cadherin (E-cad) significantly, and then affected the EMT process of tumor cells [53]. Teng et al. showed that decreased expression of HSP70 and 90 family members in prostate cancer cells leads to loss of invasion. This effect is mediated in part by controlling the critical invasion-promoting capacity of the WASF3 protein [52].

### 3.2. HSP90 Family

HSP90s are highly conserved in biological evolution with an approximately 90 kDa molecular weight, accounting for approximately 1–2% of the total protein in mammalian cells. They are molecular chaperones that promote de novo synthesis and misfolded protein folding, thereby counteracting their aggregation [9,54]. HSP90s are involved in essential cellular processes and regulatory pathways such as apoptosis, cell cycle control, protein folding and degradation, and signaling events [9]. Similar to HSP70s, HSP90s are also highly expressed in PCa and promotes the malignant progression and metastasis of PCa, which could assist in diagnosing PCa and judging patient prognosis [55,56].

#### 3.2.1. Structure of HSP90s

HSP90s function in vivo as a homodimer [57]. The HSP90 monomer consists of three highly conserved domains: (a) the amino terminal domain (NTD), which mediates binding to ATP; (b) the middle domain (MD), involved in ATP hydrolysis and HSP90s binding to client proteins; (c) the C-terminal domain (CTD), responsible for the formation of dimers (Figure 2b). The CTD also contains a C-terminal met-Glu-Glu-Val-Asp (MEEVD) motif, which is critical for interactions with co-chaperones containing tetratricopeptide repeat (TPR) domains [58]. Similar to the HSP70s, NTD and MD are connected by a long, flexible, and stretchable charged linker. This linker could regulate the binding of NTD to MD, which in turn affects the function of HSP90s [58].

#### 3.2.2. HSP90s and AR

HSP90s form a complex with AR in the cytoplasm to maintain the natural state of AR in cells, thus stabilizing AR before ligand binding. Inhibition of HSP90s leads to AR degradation and its cytoplasmic accumulation [59]. Vanaja et al. [60] treated prostate cancer cells LNCaP for 24 h with the HSP90 inhibitor geldamycin (GA) and found that GA reduced the androgen-induced AR protein level to 15%. At the same time, the expression of androgen upregulated genes such as immunophilin protein FKBP51 and prostate specific antigen (PSA) also decreased significantly. Furthermore, the maintenance of AR high affinity hormone binding conformation by HSP90s was confirmed in vitro by Fang and his colleague [61]. HSP90s push the equilibrium toward the high affinity hormone binding conformation. In this state, the binding of androgens leads to further structural changes that promote AR activation.

#### 3.2.3. HSP90s and Cell Apoptosis and Proliferation

HSP90s are widely involved in biological processes such as apoptosis, proliferation, and cycle regulation through protein folding. AC245100.4 is a LncRNA present in the cytoplasm and its association with prostate cancer was reported in previous studies [62]. Cui et al. [63] showed that AC245100.4 binds to HSP90s to alter its chaperone function by changing the levels of client proteins. When overexpressed AC245100.4, the levels of IKKα, p-IκB, p-P65, and CyclinD1 were increased, and the proliferation ability of prostate cancer cells was significantly increased. Additionally, GA could inhibit the pro-proliferative effect of AC245100.4. This suggests that AC245100.4 binding to HSP90s promotes prostate cancer progression by NF κB pathways. In addition, HSP90 can also regulate the proliferation and apoptosis of prostate cancer cells through AR, ERBB2, Akt, c-RAF, survivin, EGFR, IGFR-1, STAT3, ERK, CDK-4, and CDK-6 signaling pathways [64]. In an in vivo experiment, TRAP1 transgenic mice showed an accelerated incidence of invasive prostate adenocarcinoma characterized by increased cell proliferation and reduced cell apoptosis, and conversely, homozygous deletion of TRAP1 delayed prostate tumorigenesis in mice without affecting hyperplasia or prostate intraepithelial neoplasia [65]. This evidence suggests that HSP90s can participate in the development of prostate cancer by regulating cell proliferation and cell apoptosis.

#### 3.2.4. HSP90s and EMT, Migration, Invasion, and Metastasis

In eukaryotes, HSP90s are associated with EMT, invasion, migration, and metastasis of many tumors, such as oral cancer [66], breast cancer [67], and prostate cancer [52,68,69]. Tumor secreted extracellular HSP90 (eHSP90) initiates EMT in prostate cancer cells, promoting prostate tumor growth and invasion in vivo. Mechanistically, eHSP90 initiates sustained activation of MEK/ERK, a key signal that promotes transcriptional upregulation of EZH2 (a methyltransferase closely related to EMT) and recruitment to the E-cadherin promoter. The eHSP90-EZH2 pathway orchestrates an extended series of EMT-related events, including tumor cell migration and metastasis [68]. Additionally, eHSP90 could enhance cell motility and shifts cell morphology toward a mesenchymal phenotype in an ERK and matrix metalloproteinase-2/9-dependent manner. Conversely, inhibition of eHSP90 arrested PCa migration and shifted cell morphology towards an epithelial phenotype [69].

## 4. HSP70 Family in Prostate Cancer Treatment

In addition to hormonal therapy that removes androgen (androgen deprivation treatment (ADT)) or blocks androgen action, another important medical therapy for prostate cancer is chemotherapy mainly used for castration-resistant prostate cancer (CRPC). Radical prostatectomy remains the gold standard for PCa treatment, as drug therapies are not a radical cure even if the tumor is localized within the prostate capsule [2]. Attempts have been made over the recent decades to explore novel biomarkers of prostate cancer and develop them into novel drugs in avoidance of surgical trauma. As an important biomarker for multiple cancers, the HSP70 family has been considered as one of the promising targets that can be developed into drugs for cancer treatment. In this section, we review the agents targeting HSP70 family molecules that may be used for PCa treatment in future.

### 4.1. Small Molecular Inhibitors

Small molecular inhibitors against different HSP70 family molecules have not been successfully developed for clinical use, with only one agent being subjected to clinical trial. The first well-studied inhibitor, 2-phenylethynesulfonamide (PES) or pifithrin-µ, has been documented to bind to C-terminal SBD of HSP70 and therefore disrupt the association of HSP70s with its co-chaperone HSP40 and other client proteins such as proapoptotic ones APAF-1 and p53 [70]. This interaction of PES with HSP70 molecule induces either autophagic cell death or apoptotic, caspase-dependent, cell death, which depends on different cell types [71,72]. PES is such a potent antitumor agent in vitro and in vivo that this agent shows potent cytotoxicity in multiple cancer cells including LNCaP95 prostate cancer cells, especially when in combination with other inhibitors or medications [73,74,75].

Inhibitors that target N-terminal ATP-binding domain of HSP70s include 15-deoxyspergualin (15-DSG), VER-155008, MKT-077 (the only one subjected to clinical trial) and so on. These agents inhibit HSP70s function by attacking ATP-binding domain of HSP70 and disrupting its ATPase activity, thus blocking cellular proliferation or stimulating cell apoptosis [76,77]. 15-DSG is a natural immunosuppressive agent with a mild effect on cancerous cells compared with other inhibitors [78]. More efficacious agents are MAL3-101, the second-generation inhibitor, and its derivatives [76]. These inhibitors are minimally effective when administered alone for clinical use, but when in combination with other agents, such as HSP90 inhibitor 17-AAG and PS-341(bortezomib), they are shown to have improved efficacy [79]. VER-155008 is an adenosine-derived compound that attacks N-terminal ATP-binding domain of HSP70 family molecules. In vitro studies have demonstrated that VER-155008 is able to induce either caspase-dependent cell death or non-caspase-dependent cell death [77]. The cytotoxic effect of VER-155008 can be improved when administered in combination with HSP90 inhibitors including NVP-AUY922 [80]. The rhodacyanine dye analog MKT-077 is the only agent that has been clinically tested. MKT-077 is thought to serve as a metabolic poison in the mitochondria of cancerous cells where it induces G1 arrest and cell apoptosis [81]. However, the clinical trial of MKT-077 was halted due to the observation of nephrotoxicity, principally renal magnesium wasting. Park and his team recently reported a HSP70 inhibitor apoptosol (AZ) that inhibits the ATPase activity of HSP70s on lysosomal membrane and triggers lysosomal membrane permeabilization (LMP), ultimately inducing lysosome-mediated apoptosis of cancer cells [82,83]. This inhibitor may have higher specificity than above-mentioned HSP70 inhibitors as lysosomal HSP70s are rarely found in normal cells. The derivative of AZ, Az-TPP-O3, was also observed to have potent proapoptotic effect on cancer cells. Az-TPP-O3 primarily attacks the mitochondrial HSP70 molecule mortalin and blocks the interaction of mortalin and p53, which drives a process called mitochondrial outer membrane permeabilization (MOMP) and leads to mitochondria-mediated apoptosis due to caspase activation [82]. Unfortunately, these two unique inhibitors have not been tested in cancer patients, much less in prostate cancer patients. As a result, though all these inhibitors theoretically have strong antitumor activity, temporarily, none of them are appropriate to be developed for clinical use.

### 4.2. Immunotherapeutic Approaches

A promising strategy to inhibit the expression of HSP70s is the application of monoclonal antibodies (mAb) [79]. The mAb targets HSP70s more accurately with less side-effects than small molecular inhibitors due to its high antigen specificity. A mAb called cmHsp70.1 has completed phase I clinical trial and is being subjected to phase II trial. This monoclonal antibody can recognize the extracellular motif—TKDNNLLGRFELSG (TDK) of membrane HSP70 molecule [84]. In colon cancer, this antibody has been shown to reduce the weight and volume of tumors and promote patients’ survival when administered alone [84].

The development of HSP70 vaccines provides another immunotherapeutic approach for cancers. Several vaccines composed of disease specific epitopes and HSP70 DNA have been successfully developed and subjected to clinical trials. For example, a vaccine called pNGVL4a-Sig/E7(detox)/HSP70 DNA was clinically tested in patients with cervical intraepithelial neoplasia [85]. Another clinical trial has tested the efficacy and toxicity of another vaccine made with HSP70s in chronic myelogenous leukemia [79]. Moreover, a novel immunotherapeutic approach focusing on natural killer (NK) cells based adoptive immunotherapy is being clinically evaluated in which Hsp70-peptide TKD/IL-2 activated, autologous NK cells are employed [79]. To date, no clinical studies of HSP70-related immunotherapy have been performed in patients with prostate cancer. This must be a promising area in the exploration of PCa treatment.

### 4.3. A Novel Tool with High Specificity: Aptamers

In addition to small molecular inhibitors and antibodies, recent findings have uncovered the inhibitory effect of aptamers, a new category of targeting agents, on functions of HSP70 family molecules. Aptamers (i.e., DNA, RNA aptamers), alongside peptide aptamers, are able to bind to molecules of interest with high specificity and high affinity, exhibiting a functional similarity to antibodies. The differences between two biologics are that aptamers have relatively smaller molecular weight with decreased immunogenicity. Similar to aforesaid small molecular inhibitors, the target site of aptamers is also the NBD or SBD of HSP70 family molecules and the binding of aptamers to the target site can impair the function of HSP70s, as a result of which cancerous cells are susceptible to cell death. Screened from aptamer libraries, A17 has been considered as the most potent aptamer until now. In HeLa cells, A17 is shown to promote cell death (mainly apoptosis) and exhibit its antitumor properties in vivo when combined with cisplatin, but have no such effects when administered alone [86]. A mechanistic study has revealed that this proapoptotic effect of A17 comes from its attack on the NBD of HSP70s that disrupts HSP70 function [86]. Furthermore, the A17 aptamer can inactivate the HSP70 chaperone, but not the one of HSP90 or HSC70 [87]. All above-mentioned agents were included in a table (Table 1).

## 5. HSP90 Family in Prostate Cancer Treatment

As the most well-studied family of HSPs, HSP90 molecules and their client proteins have been shown to play fundamental roles in tumorigenesis [11]. Targeting HSP90s can lead to the proteasomal degradation of their clients including AR and ultimately influence the signal transduction related to the initiation, development and progression of cancers. This simultaneous inhibitory effects of targeting HSP90 family molecules on AR and other client proteins render HSP90 blockade an attractive therapeutic strategy for prostate cancer [64].

### 5.1. Natural HSP90 Inhibitors and Their Derivatives

The N-terminal of HSP90 molecules responsible for ATP binding is one of the binding sites for most natural HSP90 inhibitors. These molecular inhibitors ubiquitously have higher affinity for the binding site than natural nucleotides, which are able to inhibit its cycling between ATP- and ADP- bound conformations and impair the functions of HSP90 family molecules [79]. The targeting therapy using HSP90 inhibitors for cancer treatment begins with geldanamycin (GM), an agent derived from *Streptomyces hygroscopicus* with potent antitumor activity [88]. However, its phase I clinical trial was terminated due to its structural instability and hepatotoxicity. Radicicol (RD), another natural inhibitor derived from *Monosporium bonorden*, exhibits powerful in vitro antitumor properties by disrupting the critical ATP-binding site of HSP90s, but its structural instability makes it ineffective in vivo. Different from GM and RD, the natural inhibitor Novobiocin destabilizes the client proteins of HSP90 by binding to C-terminal domain of HSP90 and therefore inhibits cancer cell growth [89,90,91]. Importantly, targeting HSP90 C-terminus is one of the approaches to avoid the compensatory heat shock response, induced by targeting the HSP90 ATP-binding site, that stimulates the expression of multiple HSPs, such as HSP90, HSP70, clusterin. Unfortunately, the efficacy and safety of Novobiocin have not been rigorously tested in vivo.

The first generation GM derivatives are 17-AAG (also called tanespimycin or 17-allylamino-17-demethoxygeldanamycin) and 17-DMAG (also called as alvespimycin or 17-dimethylaminoethylamino-17-demethoxygeldanamycin). 17-AAG has been evaluated in the phase II clinical trial, but its poor water solubility and oral bioavailability restricts its development [92]. 17-DMAG shows improved solubility compared with 17-AAG; however, this agent has dose-limiting toxicities: peripheral neuropathy and renal dysfunction [93]. The next generation GM derivative, IPI-504 (retaspimycin), has been shown to have minimal efficacy and unacceptable toxicity in a phase II clinical study in CRPC patients following IPI-504 treatment [94]. This agent was therefore eliminated from therapeutic choices of prostate cancers.

The natural derivatives of RD include NVP-AUY922 and AT13387 which also attack the ATP-binding pocket of HSP90 molecules. NVP-AUY922 is the first generation RD derivative and has strong proapoptotic activity in vitro and powerful antitumor properties in an ex vivo model of prostate cancer. Nevertheless, NVP-AUY922 has not been clinically tested in patients with metastatic CRPC (mCRPC). The second generation derivative, AT13387, shows in vitro and in vivo antitumor activities against prostate cancer, with long duration of action as one of its most important characteristics [64,95]. Theoretically, the use of longer-acting HSP90 inhibitors is more likely to maintain antitumor efficacy with less systemic exposure and side effects [95]. AT13387 has been involved in multiple phase I or II clinical trials, some of which have been completed (NCT01294202, NCT01685268, NCT00878423, NCT01246102) [79]. In addition, a phase I/II clinical trial is in progress to compare the efficacy when administered alone or combined with abiraterone acetate [64]. Recent studies reported a chalcone derivative, SU086, that has potent proapoptotic effect on multiple PCa cell lines including AR-positive cell (C4-2) and AR-negative cell lines (DU145 and PC-3) [96]. This supports a notion that SU086 is active in PCa cells independence of AR status. Mechanistically, SU086 influences the glycolysis process of prostate cancer cells by impairing the function of HSP90 [97]. The clinical value of this inhibitor still needs validation by a number of clinical studies.

### 5.2. Synthetic HSP90 Inhibitors

Similar to natural inhibitors, the HSP90 N-terminus is also the important target site of synthetic HSP90 inhibitors, among which purine based compounds are one of the choices for drug development [79]. PU-H71 and PU-DZ8 are structurally analogous to ADP but have higher affinity for HSP90. This competitive effect between these two compounds and ADP hinders the binding of ADP and then impairs HSP90 functions. CNF-2024 is also a purine-scaffold compound with high oral bioavailability and has been shown to induce lymphoma cell death [98]. To date, no clinical studies have been performed to evaluate the efficacy and safety of PU series in PCa patients, therefore more clinical studies are warranted.

The screening of ATP-binding proteins using the ATP-affinity column discovered the 2-aminobenzamide derivative SNX-5422 (PF-04929113) [99]. This compound is an orally bioavailable prodrug of PF 04928473 (SNX-2112), a selective HSP90 inhibitor, and has been subjected to phase I clinical trials in patients with solid tumors including PCa as well as hematological malignancies such as lymphomas and chronic lymphocytic leukemia (CLL) [100,101,102]. Due to its ocular toxicity observed in animal models and in a phase I study, the development of SNX-5422 was terminated and waited for further evaluation [102]. Ganetespib (STA-9090), one of the RD derivatives, is a chemically synthetic compound with potent activity against prostate cancer xenografts. A phase II clinical trial, however, shows a negligible efficacy of ganetespib in 17 patients with mCRPC (only 2 patients had progression-free survival (PFS) of more than 4 months) [103]. As a compound targeting HSP90 C-terminus analogous to novobiocin, its analogue KU174 also has powerful antitumor activity against prostate cancer in vitro and in vivo without induction of the heat shock response [104]. Unfortunately, this compound has not been clinically evaluated to date. All above-mentioned inhibitors we mentioned were included in Table 2.

## 6. Conclusions

In aggregate, blocking HSP70 and HSP90 family is an emerging therapeutic target in cancer. Inhibitors and protein aptamers may be developed into various chemotherapeutic drugs while both monoclonal antibodies and vaccines are the critical parts of immunotherapies. The cytotoxicity of these agents make them able to potently reduce the size of tumors, however, only a small part of agents have been clinically tested in PCa patients showing therapeutic effects on prostate cancer. In addition, the side effect caused by these inhibitors is always not a negligible problem. Therefore, a number of clinical studies are still necessary to further evaluate the efficacy and safety of present HSP inhibitors and simultaneously the development novel agents targeting HSP70s and HSP90s will become increasingly important in the next few years.

## Figures and Tables

**Table 1:** Current agents targeting HSP70s.

Agents	Target Site	Clinical Evaluation	Reference
1. Small molecular inhibitors			
(1) PES	SBD	YES	[70,71,72,73,74,75]
(2) 15-DSG	NBD	NO	[78]
(3) MAL3-101	NBD	NO	[76,79]
(4) VER-155008	NBD	NO	[77,80]
(5) MKT-077	NBD	Halted	[81]
(6) AZ	NBD	NO	[82,83]
(7) Az-TPP-O3	NBD	NO	[82]
2. Immunotherapeutic agents			
(1) cmHsp70.1	TDK	Phase II (on going)	[84]
(2) pNGVL4a-Sig/E7(detox) /HSP70 DNA	-	Phase I	[85]
3. Aptamers			
A17	NBD	NO	[86,87]

NBD: nucleotide-binding domain; SBD: substrate-binding domain; TDK: extracellular motif—TKDNNLLGRFELSG; -: not reported.

**Table 2:** Current agents targeting HSP90s.

Agents	Targeting Site	Clinical Evaluation	Reference
1. Natural inhibitors and derivatives			
(1) GM	NTD	Halted	[88]
(2) Novobiocin	CTD	NO	[89,90,91]
(3) 17-AAG	NTD	Phase II	[92]
(4) 17-DMAG	NTD	NO	[93]
(5) IPI-504	NTD	Halted	[94]
(6) AT13387	NTD	Phase I and II	[64,79,95]
(7) SU086		NO	[96,97]
2. Synthetic inhibitors			
(1) CNF-2024	NTD	NO	[98]
(2) SNX-5422	NTD	Phase I	[99,100,101,102]
(3) STA-9090	NTD	Phase II	[103]
(4) KU174	CTD	NO	[104]

NTD: amino terminal domain; CTD: C-terminal domain; -: not reported.

## Data Availability

Not applicable.
